# Two-Sided Annihilator Condition with Generalized Derivations on Multilinear Polynomials

**DOI:** 10.1155/2014/563284

**Published:** 2014-10-28

**Authors:** V. De Filippis, G. Scudo, L. Sorrenti

**Affiliations:** Department of Mathematics, Faculty of Sciences, University of Messina, Via F. Stagno D'Alcontres 31, 98166 Messina, Italy

## Abstract

Let *R* be a prime ring of characteristic different from 2, with extended centroid *C*, *U* its two-sided Utumi quotient ring, *F* a nonzero generalized derivation of *R*, *f*(*x*_1_,…, *x*_*n*_) a noncentral multilinear polynomial over *C* in *n* noncommuting variables, and *a*, *b* ∈ *R* such that *a*[*F*(*f*(*r*_1_,…, *r*_*n*_)), *f*(*r*_1_,…, *r*_*n*_)]*b* = 0 for any *r*_1_,…, *r*_*n*_ ∈ *R*. Then one of the following holds: (1) *a* = 0; (2) *b* = 0; (3) there exists *λ* ∈ *C* such that *F*(*x*) = *λx*, for all *x* ∈ *R*; (4) there exist *q* ∈ *U* and *λ* ∈ *C* such that *F*(*x*) = (*q* + *λ*)*x* + *xq*, for all *x* ∈ *R*, and *f*(*x*_1_,…, *x*_*n*_)^2^ is central valued on *R*; (5) there exist *q* ∈ *U* and *λ*, *μ* ∈ *C* such that *F*(*x*) = (*q* + *λ*)*x* + *xq*, for all *x* ∈ *R*, and *aq* = *μa*, *qb* = *μb*.

## 1. Introduction

Let *R* be a prime ring with center *Z*(*R*). We denote by [*a*, *b*] = *ab* − *ba* the simple commutator of the elements *a*, *b* ∈ *R* and by [*a*, *b*]_*k*_ = [[*a*, *b*]_*k*−1_, *b*], for *k* > 1, the *k*th commutator of *a*,  *b*. Throught this paper we will use the following notation: *U* will be the (two-sided) Utumi quotient ring of a ring *R* (sometimes, as in [1], *U* is called the symmetric ring of quotients). The definition, the axiomatic formulation, and the properties of this quotient ring *U* can be found in [1–3].

In any case, when *R* is a prime ring, all that we need here about this object is that

(1) *R*⊆*U*;
(2) *U* is a prime ring;
(3) the center of *U*, denoted by *C* is a field which is called the extended centroid of *R*.

A well known result of Posner [4] says that if *d* is a derivation of *R* such that [*d*(*x*), *x*] ∈ *Z*(*R*), for all *x* ∈ *R*, then *R* is commutative. In [5] Lanski generalizes the result of Posner, by replacing the element *x* ∈ *R* with an element of a noncentral Lie ideal *L* of *R*. More precisely he proves that if [*d*(*x*), *x*]_*k*_ = 0 for all *x* ∈ *L* and *k* ≥ 1 a fixed integer; then char(*R*) = 2 and *R* satisfies *s*_4_, the standard identity of degree 4.

Let *f*(*x*_1_,…, *x*_*n*_) be a multilinear polynomial over *C* in *n* noncommuting variables and denote by *f*(*X*) the set of all evaluations of *f*(*x*_1_,…, *x*_*n*_) in *X*⊆*R*. In case *f*(*x*_1_,…, *x*_*n*_) is not central valued on *R*, it is well known that the additive subgroup generated by *f*(*R*) contains a noncentral Lie ideal of *R*. Moreover any noncentral Lie ideal of *R* contains all the commutators [*x*, *y*] for *x*, *y* in some nonzero ideal of *R*, unless char(*R*) = 2 and dim_*C*_ *RC* = 4.

In light of this and following the line of investigation of the previous cited papers, in [6] P. H. Lee and T. K. Lee consider the Engel-condition [*d*(*x*), *x*]_*k*_ = 0, in case *x* ∈ *f*(*I*), where *I* is a two-sided ideal of *R*. They show that either *f*(*x*_1_,…, *x*_*n*_) is central valued in *R* or char(*R*) = 2 and *R* satisfies *s*_4_.

These results indicate that the global structure of a prime ring *R* is often tightly connected to the behaviour of additive mappings defined on *R*, which act on suitable subsets of the whole ring. In [7] de Filippis and di Vincenzo study the left annihilator of the set {*d*(*u*)*u* − *ud*(*u*),  *u* ∈ *f*(*R*)}, where *d* is a derivation. In case the annihilator is not zero, the conclusion is that *f*(*x*_1_,…, *x*_*n*_) is central valued on *R*. These facts in a prime ring are natural tests which evidence that the set {*d*(*u*)*u* − *ud*(*u*),  *u* ∈ *f*(*R*)} is rather large in *R*.

More recently, Liu [8] and Wang [9] have examined the identity *a*[*d*(*u*), *u*]_*k*_ = 0, where *d* is a derivation of *R* and *u* ∈ *f*(*I*), where *I* is a one-sided ideal of *R*. In particular, for *I* = *R*, if *a* ≠ 0 and *f*(*x*_1_,…, *x*_*n*_) is not central valued on *R*, then char(*R*) = 2 and *R* satisfies *s*_4_.

In [10] de Filippis considers a similar situation, in the case the derivation *d* is replaced by a generalized derivation *F*. An additive map *F* : *R* → *R* is said to be a generalized derivation if there is a derivation *d* of *R* such that, for all *x*, *y* ∈ *R*, *F*(*xy*) = *F*(*x*)*y* + *xd*(*y*). A significative example is a map of the form *F*(*x*) = *ax* + *xb*, for some *a*, *b* ∈ *R*; such generalized derivations are called inner. Generalized derivations have been primarily studied on operator algebras. Therefore any investigation from the algebraic point of view might be interesting (see, e.g., [11]).

The main result in [10] is the following.

**Theorem 1 A.**

Let *R* be a prime ring of characteristic different from 2, with extended centroid *C*, *U* its two-sided Utumi quotient ring, *F* ≠ 0 a nonzero generalized derivation of *R*, *f*(*x*_1_,…, *x*_*n*_) a noncentral multilinear polynomial over *C* in n noncommuting variables, and *a* ∈ *R* such that

(1) $$\begin{array}{c} a\left[F\left(f\left(r_{1},\ldots ,r_{n}\right)\right),f\left(r_{1},\ldots ,r_{n}\right)\right]=0 \end{array}$$

for any *r*_1_,…, *r*_*n*_ ∈ *R*. Then either *a* = 0 or one of the following holds:

(1) there exists *λ* ∈ *C* such that *F*(*x*) = *λx*, for all *x* ∈ *R*;
(2) there exist *q* ∈ *U* and *λ* ∈ *C* such that *F*(*x*) = (*q* + *λ*)*x* + *xq*, for all *x* ∈ *R*, and *f*(*x*_1_,…, *x*_*n*_)^2^ is central valued on *R*.

We would like to remark that the same conclusions hold in case we consider the right annihilator, more precisely.

**Theorem 1 B.**

Let *R* be a prime ring of characteristic different from 2, with extended centroid *C*, *U* its two-sided Utumi quotient ring, *F* ≠ 0 a nonzero generalized derivation of *R*, *f*(*x*_1_,…, *x*_*n*_) a noncentral multilinear polynomial over *C* in n noncommuting variables, and *a* ∈ *R* such that

(2) $$\begin{array}{c} \left[F\left(f\left(r_{1},\ldots ,r_{n}\right)\right),f\left(r_{1},\ldots ,r_{n}\right)\right]a=0 \end{array}$$

for any *r*_1_,…, *r*_*n*_ ∈ *R*. Then either *a* = 0 or one of the following holds:

(1) there exists *λ* ∈ *C* such that *F*(*x*) = *λx*, for all *x* ∈ *R*;
(2) there exist *q* ∈ *U* and *λ* ∈ *C* such that *F*(*x*) = (*q* + *λ*)*x* + *xq*, for all *x* ∈ *R*, and *f*(*x*_1_,…, *x*_*n*_)^2^ is central valued on *R*.

Here we will consider a more general situation, involving a two-sided annihilating condition. More specifically, we study simultaneously left and right annihilators of the set {[*F*(*x*), *x*] : *x* ∈ *f*(*R*)} and prove the following.

**Theorem 1 .**

Let *R* be a prime ring of characteristic different from 2, with extended centroid *C*, *U* its two-sided Utumi quotient ring, *F* a nonzero generalized derivation of *R*, *f*(*x*_1_,…, *x*_*n*_) a noncentral multilinear polynomial over *C* in n noncommuting variables, and *a*, *b* ∈ *R* such that

(3) $$\begin{array}{c} a\left[F\left(f\left(r_{1},\ldots ,r_{n}\right)\right),f\left(r_{1},\ldots ,r_{n}\right)\right]b=0 \end{array}$$

for any *r*_1_,…, *r*_*n*_ ∈ *R*. Then one of the following holds:

(1) *a* = 0;
(2) *b* = 0;
(3) there exists *λ* ∈ *C* such that *F*(*x*) = *λx*, for all *x* ∈ *R*;
(4) there exist *q* ∈ *U* and *λ* ∈ *C* such that *F*(*x*) = (*q* + *λ*)*x* + *xq*, for all *x* ∈ *R*, and *f*(*x*_1_,…, *x*_*n*_)^2^ is central valued on *R*;
(5) there exist *q* ∈ *U* and *λ*, *μ* ∈ *C* such that *F*(*x*) = (*q* + *λ*)*x* + *xq*, for all *x* ∈ *R*, and *aq* = *μa*, *qb* = *μb*.

**Remark 2 .**

By the primeness of *R* and in light of Theorems A and B, we may assume that *R* is not a domain. Moreover, since the center of a prime ring cannot contain nonzero zero-divisor, then neither *a* ∈ *Z*(*R*) nor *b* ∈ *Z*(*R*). Finally in all that follows we always suppose char(*R*) ≠ 2.

In the sequel we will make a frequent use of the following.

**Remark 3 .**

If *B* is a basis of *U* over *C* then any element of *T* = *U*∗_*C*_ *C*{*x*_1_,…, *x*_*n*_}, the free product over *C* of the *C*-algebra *U* and the free *C*-algebra *C*{*x*_1_,…, *x*_*n*_}, is called generalized polynomial and can be written in the form *g* = ∑_*i*_ *α*_*i*_ *m*_*i*_. In this decomposition the coefficients *α*_*i*_ are in *C* and the elements *m*_*i*_ are *B*-monomials; that is, *m*_*i*_ = *q*_0_ *y*_1_ *q*_1_ ⋯ *y*_*h*_ *q*_*h*_, with *q*_*i*_ ∈ *B* and *y*_*i*_ ∈ {*x*_1_,…, *x*_*n*_}. In [12] it is shown that a generalized polynomial *g* = ∑_*i*_ *α*_*i*_ *m*_*i*_ is the zero element of *T* if and only if all *α*_*i*_ are zero. Let *a*_1_,…, *a*_*k*_ ∈ *U* be linearly independent over *C* and *a*_1_ *g*_1_(*x*_1_,…, *x*_*n*_)+⋯+*a*_*k*_ *g*_*k*_(*x*_1_,…, *x*_*n*_) = 0 ∈ *T*, for some *g*_1_,…, *g*_*k*_ ∈ *T*. If, for any *i*, *g*_*i*_(*x*_1_,…, *x*_*n*_) = ∑_*j*=1_^*n*^ *x*_*j*_ *h*_*j*_(*x*_1_,…, *x*_*n*_) and *h*_*j*_(*x*_1_,…, *x*_*n*_) ∈ *T*, then *g*_1_(*x*_1_,…, *x*_*n*_),…, *g*_*k*_(*x*_1_,…, *x*_*n*_) are the zero element of *T*. The same conclusion holds if *g*_1_(*x*_1_,…, *x*_*n*_)*a*_1_ + ⋯+*g*_*k*_(*x*_1_,…, *x*_*n*_)*a*_*k*_ = 0 ∈ *T*, and *g*_*i*_(*x*_1_,…, *x*_*n*_) = ∑_*j*=1_^*n*^ *h*_*j*_(*x*_1_,…, *x*_*n*_)*x*_*j*_ for some *h*_*j*_(*x*_1_,…, *x*_*n*_) ∈ *T*.

We refer the reader to [1, 12] for more details on generalized polynomial identities.

## 2. An Independent Result

We will dedicate this section to the proof of the following proposition on linear identities with commutators in matrix rings. This result will be useful in the sequel.

**Proposition 4 .**

Let *C* be a field and *R* = *M*_*t*_(*C*) the algebra of *t* × *t* matrices over *C* and *S* = [*R*, *R*]. Let *a*, *b*, *c* ∈ *R*, such that *c* ∉ *Z*(*R*) and *a*[*c*, *x*]*b* = 0 for all *x* ∈ *S*. Then there exists *λ* ∈ *Z*(*R*) such that *ac* = *λa* and *cb* = *λb*.

In order to prove Proposition 4, we need several lemmas.

**Lemma 5 .**

Let *K* be an infinite field and *t* ≥ 2. If *A*_1_,…, *A*_*k*_ are not scalar matrices in *M*_*t*_(*K*), then there exists some invertible matrix *B* ∈ *M*_*t*_(*K*) such that each matrix *BA*_1_ *B* ^−1^,…, *BA*_*k*_ *B* ^−1^ has all nonzero entries.

**Proof**

See Lemma  1.5 in [13].

**Lemma 6 .**

Let *R* be a prime ring with extended centroid *C*. Suppose ∑_*i*=1_^*n*^ *a*_*i*_ *xb*_*i*_ + ∑_*j*=1_^*m*^ *c*_*j*_ *xd*_*j*_ = 0, for all *x* ∈ *R*, where *a*_*i*_, *b*_*i*_, *c*_*j*_, *d*_*j*_ ∈ *R*, for *i* = 1,…, *n* and *j* = 1,…, *m*. If *a*_1_,…, *a*_*n*_ are *C*-independent then each *b*_*i*_ is *C*-dependent on *d*_1_,…, *d*_*m*_. Analogously if *b*_1_,…, *b*_*n*_ are *C*-independent then each *a*_*i*_ is *C*-dependent on *c*_1_,…, *c*_*m*_.

**Proof**

It is Martindale's result contained in [14].

**Lemma 7 .**

Let *R* be a prime ring with extended centroid *C*. Suppose *a*[*x*, *y*]+[*x*, *y*]*b* = 0, for all *x*, *y* ∈ *R*, where *a*, *b* ∈ *R*. Then *a* = −*b* ∈ *C*.

**Proof**

It is an easy consequence of Lemma 6.

**Lemma 8 .**

Let *K* be an infinite field, *R* = *M*_*m*_(*K*) the algebra of *m* × *m* matrices over *K*, *Z*(*R*) the center of *R*, and *S* = [*R*, *R*]. Assume that there exist *a*,  *b*,  *c*,  *q* nonzero elements of *R* such that *axq* + *cxb* = 0 for all *x* ∈ *S*. If *q* ∈ *Z*(*R*) then one of the following holds:

(1) *a*,  *b*,  *c* are central matrices and *aq* + *bc* = 0;
(2) *b* is a central matrix and *aq* + *bc* = 0.

**Proof**

Since *q* ∈ *Z*(*R*), by the assumption, we have that *aqx* + *cxb* = 0 for all *x* ∈ *S*. Clearly if *c* ∈ *Z*(*R*) then *aqx* + *xbc* = 0 for all *x* ∈ *S*, and by Lemma 7 we get *aq* = −*bc* ∈ *Z*(*R*); that is, *a*, *b*, *c* ∈ *Z*(*R*). On the other hand, if *b* ∈ *Z*(*R*), then (*aq* + *bc*)*x* = 0 for all *x* ∈ *S* and it follows easily that *aq* + *bc* = 0.

In light of this, we consider *c* and *b* both nonscalar matrices. We will prove that in this case we get a contradiction.

Here we denote by *e*_*ij*_ the usual matrix unit with 1 in the (*i*, *j*)-entry and zero elsewhere.

By Lemma 5, we can assume that *c* and *b* have all nonzero entries, say *c* = ∑_*kl*_ *c*_*kl*_ *e*_*kl*_ and *b* = ∑_*kl*_ *b*_*kl*_ *e*_*kl*_, for 0 ≠ *c*_*kl*_, 0 ≠ *b*_*kl*_ ∈ *K*.

Since *e*_*ji*_ ∈ *S* for all *i* ≠ *j*, then, for any *i* ≠ *j*,

(4) $$\begin{array}{c} X=aqe_{ji}+ce_{ji}b=0 \end{array}$$

in particular the (*i*, *j*)-entry of *X* is *c*_*ij*_ *b*_*ij*_ = 0, a contradiction.

For sake of clearness, we may write the previous lemma as follows.

**Lemma 9 .**

Let *K* be an infinite field, *R* = *M*_*m*_(*K*) the algebra of *m* × *m* matrices over *K*, *Z*(*R*) the center of *R*, and *S* = [*R*, *R*]. Let *a*_1_,  *a*_2_,  *a*_3_,  *a*_4_ be nonzero elements of *R* such that *a*_1_ *xa*_2_ + *a*_3_ *xa*_4_ = 0 for all *x* ∈ *S*. Assume there exists *i* ∈ {1,2, 3,4} such that *a*_*i*_ ∈ *Z*(*R*). Then *a*_1_ = *αa*_3_ and *a*_2_ = −*αa*_4_, for a suitable *α* ∈ *Z*(*R*).

**Lemma 10 .**

Let *K* be an infinite field, *R* = *M*_*m*_(*K*) the algebra of *m* × *m* matrices over *K*, and *Z*(*R*) the center of *R*. Assume that there exist *a*,  *b*,  *c*,  *q* nonzero elements of *R* such that *axq* + *cxb* = 0 for all *x* ∈ *S* = [*R*, *R*]. If *q* ∉ *Z*(*R*) and *b* − *αq* ∈ *Z*(*R*), for a suitable *α* ∈ *K*, then *b* − *αq* = *a* + *αc* = 0.

**Proof**

Assume that *a* + *αc* is not a scalar matrix. By Lemma 5, we can assume that *a* + *αc* and *q* have all nonzero entries, say *a* + *αc* = ∑_*kl*_ *t*_*kl*_ *e*_*kl*_ and *q* = ∑_*kl*_ *q*_*kl*_ *e*_*kl*_, for 0 ≠ *t*_*kl*_, 0 ≠ *q*_*kl*_ ∈ *K*.

Since *b* = *βI* + *αq*, for a suitable *β* ∈ *K*, by our assumption we have that

(5) $$\begin{array}{c} axq+cx\left(\beta +\alpha q\right)=0; \end{array}$$

that is,

(6) $$\begin{array}{c} \beta cx+\left(a+\alpha c\right)xq=0, \end{array}$$

for all *x* ∈ *S*. In particular for *x* = [*e*_*ii*_, *e*_*ij*_] = *e*_*ij*_, with *i* ≠ *j*,

(7) $$\begin{array}{c} 0=X=\beta ce_{ij}+\left(a+\alpha c\right)e_{ij}q. \end{array}$$

By calculations one has that the (*j*, *i*)-entry of *X* is 0 = *t*_*ji*_ *q*_*ji*_, a contradiction.

Therefore *a* + *αc* must be a central matrix. In light of this, there exist *β*, *γ* ∈ *K* such that *b* = *αq* + *β* and *a* = −*αc* + *γ*, so that 0 = (−*αc* + *γ*)*xq* + *cx*(*αq* + *β*) = (*βc*)*x* + *x*(*γq*), for all *x* ∈ *S* = [*R*, *R*]. Once again by Lemma 7 and since *q* ∉ *Z*(*R*), it follows that *β* = *γ* = 0; that is, *b* = *αq* and *a* = −*αc*.

**Lemma 11 .**

Let *K* be an infinite field, *R* = *M*_*m*_(*K*) the algebra of *m* × *m* matrices over *K*, and *S* = [*R*, *R*]. Suppose there exist *a*,  *b*,  *c*,  *q* ∈ *R* such that *axq* + *cxb* = 0 for all *x* ∈ *S*. Denote

(8) $$\begin{array}{c} a=\underset{kl}{\sum }a_{kl}e_{kl},\;\;b=\underset{kl}{\sum }b_{kl}e_{kl},\\ c=\underset{kl}{\sum }c_{kl}e_{kl},\;\;q=\underset{kl}{\sum }q_{kl}e_{kl} \end{array}$$

for suitable *a*_*kl*_,  *b*_*kl*_,  *c*_*kl*_, and *q*_*kl*_ elements of *K*. If there are *i* ≠ *j* such that *q*_*ji*_ ≠ 0, *c*_*ji*_ ≠ 0, and *b*_*ji*_ = 0, then *a*_*ri*_ = 0 and *b*_*rk*_ = 0 for all *r* ≠ *i* and *k* ≠ *r* (i.e., the only nonzero off-diagonal elements of *b* fall in the *i*th row).

**Proof**

Consider the assumption

(9) $$\begin{array}{c} axq+cxb=0\;\forall x\in \left[R,R\right]. \end{array}$$

In particular, for *x* = *e*_*ij*_, we have

(10) $$\begin{array}{c} X=ae_{ij}q+ce_{ij}b=0 \end{array}$$

so that, for all *r* ≠ *i*, the (*r*, *i*)-entry of the matrix *X* is 0 = *a*_*ri*_ *q*_*ji*_ + *c*_*ri*_ *b*_*ji*_ = *a*_*ri*_ *q*_*ji*_. Since *q*_*ji*_ ≠ 0, one has *a*_*ri*_ = 0 for all *r* ≠ *i*, in particular *a*_*ji*_ = 0. Thus, in case *m* = 2 we are done (since *b*_*ji*_ = *a*_*ji*_ = 0).

Assume in what follows that *m* ≥ 3, and choose *x* = *e*_*it*_, with *t* ≠ *i*, *j*. Hence we also have

(11) $$\begin{array}{c} Y=ae_{it}q+ce_{it}b=0. \end{array}$$

From the previous equalities it follows that

(1) for all *s* ≠ *i*, the (*j*, *s*)-entry of the matrix *X* is *a*_*ji*_ *q*_*js*_ + *c*_*ji*_ *b*_*js*_ = 0;
(2) for all *s* ≠ *i*, *j*, the (*j*, *s*)-entry of the matrix *Y* is *a*_*ji*_ *q*_*ts*_ + *c*_*ji*_ *b*_*ts*_ = 0;
(3) the (*j*, *i*)-entry of the matrix *Y* is *a*_*ji*_ *q*_*ti*_ + *c*_*ji*_ *b*_*ti*_ = 0;
(4) for all *k* ≠ *i*, *t*, the (*j*, *k*)-entry of the matrix *Y* is *a*_*ji*_ *q*_*tk*_ + *c*_*ji*_ *b*_*tk*_ = 0 (note that this holds also in case *k* = *j*).

By (1) and (2) and since *a*_*ji*_ = 0 and *c*_*ji*_ ≠ 0, we have both *b*_*js*_ = 0, for all *s* ≠ *i*, and *b*_*ts*_ = 0 for all *t* ≠ *i*, *j* and *s* ≠ *i*, *j*. So by (3)  *b*_*ti*_ = 0 for all *t* ≠ *i*. Finally by (4), *b*_*tk*_ = 0 for all *t* ≠ *i*, *j* and *k* ≠ *t*.

**Lemma 12 .**

Let *K* be an infinite field, *R* = *M*_*m*_(*K*) the algebra of *m* × *m* matrices over *K*, and *S* = [*R*, *R*]. Suppose there exist *a*, *b*, *c*, *q* ∈ *R* such that *axq* + *cxb* = 0 for all *x* ∈ *S*. Denote

(12) $$\begin{array}{c} b=\underset{kl}{\sum }b_{kl}e_{kl},\;\;c=\underset{kl}{\sum }c_{kl}e_{kl},\;\;q=\underset{kl}{\sum }q_{kl}e_{kl} \end{array}$$

for suitable *b*_*kl*_, *c*_*kl*_, and *q*_*kl*_ elements of *K*. Assume there are *i* ≠ *j* such that *b*_*ji*_ = 0. If *q*_*rs*_ ≠ 0, *c*_*rs*_ ≠ 0 for all *r* ≠ *s*, then one of the following holds:

(1) *a* = *b* = 0;
(2) *m* = 2, *cq* = 0, and there exists 0 ≠ *λ* ∈ *K* such that
  (13) $$\begin{array}{c} a=\left[\begin{array}{cc} 0 & \lambda c_{12}\\ 0 & \lambda c_{22} \end{array}\right],\;\;b=\left[\begin{array}{cc} -\lambda q_{11} & -\lambda q_{12}\\ 0 & 0 \end{array}\right]. \end{array}$$

**Proof**

Firstly we consider the case *m* ≥ 3. The first step is to apply twice Lemma 11: this forces *b* to be a diagonal matrix. In fact *b*_*ji*_ = 0, *q*_*ji*_ ≠ 0, and *c*_*ji*_ ≠ 0 imply that *b*_*rk*_ = 0 for all *r* ≠ *i* and *k* ≠ *r*; in particular, since *m* ≥ 3, there exists *t* ≠ *i* such that *b*_*lt*_ = 0, for all *l* ≠ *t*, *i*. Since *q*_*lt*_ ≠ 0, *c*_*lt*_ ≠ 0, we have *b*_*rk*_ = 0 for all *r* ≠ *t* and *k* ≠ *r*, so *b*_*ik*_ = 0 for all *k* ≠ *i*, as required. Say *b* = ∑_*k*_ *b*_*kk*_ *e*_*kk*_.

Consider now the inner automorphism of *R* induced by the invertible matrix *P* = *I* + *e*_*rj*_, for *r* ≠ *i*, *j*: *φ*(*x*) = *P* ^−1^ *xP*. Of course *φ*(*a*)*xφ*(*q*) + *φ*(*c*)*xφ*(*b*) = 0, for all *x* ∈ *S*. Moreover the (*j*, *i*)-entries of *φ*(*q*), *φ*(*c*), and *φ*(*b*) are, respectively, *q*_*ji*_ ≠ 0, *c*_*ji*_ ≠ 0, and *b*_*ji*_ = 0. Therefore, again by Lemma 11, any (*r*, *j*)-entry of *φ*(*b*) is zero, for all *r* ≠ *i*. By calculations 0 = (*φ*(*b*))_*rj*_ = *b*_*jj*_ − *b*_*rr*_; that is, *b*_*jj*_ = *b*_*rr*_.

On the other hand, if *χ* is the inner automorphisms induced by the invertible matrix *Q* = *I* + *e*_*ri*_, as above *χ*(*a*)*xχ*(*q*) + *χ*(*c*)*xχ*(*b*) = 0, for all *x* ∈ *R*. Since the (*i*, *j*)-entries of *χ*(*q*), *χ*(*c*), and *χ*(*b*) are, respectively, *q*_*ij*_ ≠ 0, *c*_*ij*_ ≠ 0, and *b*_*ij*_ = 0, and again any (*r*, *i*)-entry of *χ*(*b*) is zero, for all *r* ≠ *j*; that is, 0 = (*φ*(*b*))_*ri*_ = *b*_*ii*_ − *b*_*rr*_ and *b*_*ii*_ = *b*_*rr*_ = *b*_*jj*_ = *β*, for all *r* ≠ *i*, *j*. Thus *b* = *βI* is a central matrix in *R*. By Lemma 9, either *b* = *αq* for some *α* ∈ *K* or *b* = 0. Since the first case cannot occur, we get *b* = 0 and also *a* = 0 which follows from *a*[*R*, *R*]*q* = 0 and *q* ≠ 0.

Let now *m* = 2; that is, *R* = *M*_2_(*K*). In this case it is well known that for any element *x* ∈ [*R*, *R*] there exist *α*, *β*, *γ* ∈ *K* such that $x=\left[\begin{array}{cc} \alpha & \beta \\ \gamma & -\alpha \end{array}\right]$. Without loss of generality we may assume *b*_21_ = 0. In case *b*_12_ = 0, then by the same above argument we show that *b* ∈ *Z*(*R*) and we are done again. Thus we consider the case *b*_12_ ≠ 0. Moreover, by applying Lemma 11 it follows *a*_21_ = 0. Hence we may write

(14) $$\begin{array}{c} a=\left[\begin{array}{cc} a_{11} & a_{12}\\ 0 & a_{22} \end{array}\right],\;\;b=\left[\begin{array}{cc} b_{11} & b_{12}\\ 0 & b_{22} \end{array}\right]. \end{array}$$

For *x* = *e*_12_ ∈ [*R*, *R*] we have

(15) $$\begin{array}{c} X=ae_{12}q+ce_{12}b=0 \end{array}$$

so that the (2,2)-entry of the matrix *X* is 0 = *c*_21_ *b*_22_; that is, *b*_22_ = 0 and the (1,1)-entry of the matrix *X* is 0 = *a*_11_ *q*_21_; that is, *a*_11_ = 0. On the other hand, for *x* = *e*_21_ ∈ [*R*, *R*], we have

(16) $$\begin{array}{c} Y=ae_{21}q+ce_{21}b=0. \end{array}$$

The (1,2)-entry of the matrix *Y* is 0 = *a*_12_ *q*_12_ + *c*_12_ *b*_12_; that is, *a*_12_ ≠ 0 and *b*_12_/*q*_12_ = −*a*_12_/*c*_12_. Moreover the (2,2)-entry of the matrix *Y* is 0 = *a*_22_ *q*_12_ + *c*_22_ *b*_12_. Therefore, if denoted *λ* = −*b*_12_/*q*_12_, one has *a*_22_ = *λc*_22_ and *a*_12_ = *λc*_12_.

Analogously, the (1,1)-entry of the matrix *Y* is 0 = *a*_12_ *q*_11_ + *c*_12_ *b*_11_. Thus *b*_11_ = −*λq*_11_ and *b*_12_ = −*λq*_12_.

Finally, by our assumption and for $x=\left[\begin{array}{cc} \alpha & \beta \\ \gamma & -\alpha \end{array}\right]$, with *α* ≠ 0, we also have

(17) 0λc120λc22·αβγ−α·q11q12q21q22  +c11c12c21c22·αβγ−α·−λq11−λq1200=0,

and by easy calculations it follows *cq* = 0.

**Lemma 13 .**

Let *K* be an infinite field, *R* = *M*_2_(*K*) the algebra of *m* × *m* matrices over *K*, and *S* = [*R*, *R*]. Let *a*, *b*, *c* ∈ *R* and denote

(18) $$\begin{array}{c} a=\underset{kl}{\sum }a_{kl}e_{kl},\;\;b=\underset{kl}{\sum }b_{kl}e_{kl},\\ c=\underset{kl}{\sum }c_{kl}e_{kl},\;\;cb=\underset{kl}{\sum }p_{kl}e_{kl} \end{array}$$

for suitable *a*_*kl*_, *b*_*kl*_, *c*_*kl*_, and *p*_*kl*_ elements of *K*. Suppose *c* ∉ *Z*(*R*) and *a*[*c*, *x*]*b* = 0 for all *x* ∈ *S*. Assume there are *i* ≠ *j* such that *p*_*ji*_ = 0. If *a*_*rs*_ ≠ 0, *b*_*rs*_ ≠ 0, for all *r* ≠ *s*, then *ac* = *cb* = 0.

**Proof**

By our hypothesis, we have *acxb* − *axcb* = 0 for all *x* ∈ *S*. By Lemma 12 it follows that either *ac* = *cb* = 0 or *ab* = 0 and there exists 0 ≠ *λ* ∈ *K* such that

(19) $$\begin{array}{c} ac=\left[\begin{array}{cc} 0 & \lambda a_{12}\\ 0 & \lambda a_{22} \end{array}\right],\;\;cb=\left[\begin{array}{cc} \lambda b_{11} & \lambda b_{12}\\ 0 & 0 \end{array}\right]. \end{array}$$

Notice that *ab* = 0 implies that the following holds:

(20) $$\begin{array}{c} a_{11}b_{11}+a_{12}b_{21}=0, \end{array}$$

(21) $$\begin{array}{c} a_{21}b_{11}+a_{22}b_{21}=0. \end{array}$$

Moreover, by computing the product *ac* we get

(22) $$\begin{array}{c} a_{11}c_{11}+a_{12}c_{21}=0, \end{array}$$

(23) $$\begin{array}{c} a_{21}c_{11}+a_{22}c_{21}=0, \end{array}$$

(24) $$\begin{array}{c} a_{11}c_{12}+a_{12}c_{22}=\lambda a_{12}. \end{array}$$

Finally, by computing the product *cb* we also have

(25) $$\begin{array}{c} c_{21}b_{11}+c_{22}b_{21}=0, \end{array}$$

(26) $$\begin{array}{c} c_{21}b_{12}+c_{22}b_{22}=0. \end{array}$$

Notice that, in case *a*_11_ = 0, by (20) it follows the contradiction *a*_12_ *b*_21_ = 0. Thus *a*_11_ ≠ 0 and multiply (25) by *a*_11_, so that *c*_21_ *b*_11_ *a*_11_ + *c*_22_ *b*_21_ *a*_11_ = 0. Again by (20) we have *b*_21_(*c*_22_ *a*_11_ − *c*_21_ *a*_12_) = 0 and using (22) it follows *b*_21_(*c*_22_ *a*_11_ + *c*_11_ *a*_11_) = 0. Since *b*_21_ ≠ 0 and *a*_11_ ≠ 0, then *c*_11_ = −*c*_22_ = *μ*.

Assume *μ* ≠ 0, denoted by *I* the identity matrix in *R*, and let $c^{\text{'}}=c-\mu I=\left[\begin{array}{cc} 0 & c_{12}\\ c_{21} & -2\mu \end{array}\right]$.

Since *c* and *c*′ induce the same inner derivation, then by our assumptions we have that *a*[*c*′, *x*]*b* = 0 for all *x* ∈ *S*. By applying again Lemma 12, it follows that either *ac*′ = *c*′*b* = 0 or *ab* = 0 and there exists 0 ≠ *ν* ∈ *K* such that

(27) $$\begin{array}{c} ac^{\text{'}}=\left[\begin{array}{cc} 0 & \nu a_{12}\\ 0 & \nu a_{22} \end{array}\right],\;\;c^{\text{'}}b=\left[\begin{array}{cc} \nu b_{11} & \nu b_{12}\\ 0 & 0 \end{array}\right]. \end{array}$$

In the latter case, by using the same above argument, the matrix *c*′ satisfies the equalities (22) and (25); that is, respectively,

(28) $$\begin{array}{c} a_{12}c_{21}=0 \end{array}$$

implying *c*_21_ = 0, and

(29) $$\begin{array}{c} -2\mu b_{21}=0 \end{array}$$

which is a contradiction.

Therefore

(30) $$\begin{array}{c} ac^{\text{'}}=c^{\text{'}}b=0,\;\text{i.e.,}\;\;ac=\mu a,\;\;cb=\mu b. \end{array}$$

In this case, by using both (22) and (30), the (1,1)-entry of the matrix *ac* should be

(31) $$\begin{array}{c} 0=a_{11}c_{11}+a_{12}c_{21}=\mu a_{11}\neq 0. \end{array}$$

The previous contradiction implies *μ* = 0; that is, *c*_22_ = 0 and by (26) also *c*_21_ = 0. Hence $c=\left[\begin{array}{cc} 0 & c_{12}\\ 0 & 0 \end{array}\right]$.

Now consider the following elements in *S*:

(32) $$\begin{array}{c} x_{0}=\left[\begin{array}{cc} 1 & 1\\ -1 & -1 \end{array}\right],\;\;y_{0}=\left[\begin{array}{cc} 1 & -1\\ 1 & -1 \end{array}\right]. \end{array}$$

Thus

(33) $$\begin{array}{c} Z=a\left[c,x_{0}\right]b=0\;\;T=a\left[c,y_{0}\right]b=0 \end{array}$$

and in particular the (1,1)-entry of *Z* is

(34) $$\begin{array}{c} c_{12}\left(-a_{11}b_{11}-2a_{11}b_{21}+a_{21}b_{21}\right)=0, \end{array}$$

and the (1,1)-entry of *T* is

(35) $$\begin{array}{c} c_{12}\left(a_{11}b_{11}-2a_{11}b_{21}-a_{21}b_{21}\right)=0. \end{array}$$

Since *c* ≠ 0, then *c*_12_ ≠ 0. Therefore the sum of (34) and (35) forces the contradiction −4*a*_11_ *b*_21_ = 0.

**Lemma 14 .**

Let *K* be an infinite field, *R* = *M*_*t*_(*K*) the algebra of *t* × *t* matrices over *K*, and *S* = [*R*, *R*]. Let *a*, *b*, *c* ∈ *R* and denote

(36) $$\begin{array}{c} a=\underset{kl}{\sum }a_{kl}e_{kl},\;\;b=\underset{kl}{\sum }b_{kl}e_{kl},\\ cb=\underset{kl}{\sum }p_{kl}e_{kl},\;\;ac=\underset{kl}{\sum }q_{kl}e_{kl} \end{array}$$

for suitable *a*_*kl*_, *b*_*kl*_, *p*_*kl*_, and *q*_*kl*_ elements of *K*. Suppose *c* ∉ *Z*(*R*) and *a*[*c*, *x*]*b* = 0 for all *x* ∈ *S*. Then there exists *λ* ∈ *Z*(*R*) such that *ac* = *λa* and *cb* = *λb*.

**Proof**

Clearly if one of *a*, *b*, *ac*, or *cb* is a scalar matrix we are done by Lemma 9. In order to prove this lemma, we may assume that *a*, *b*, *ac*, and *cb* are noncentral matrices.

By Lemma 5, there exists some invertible matrix *Q* ∈ *M*_*t*_(*K*) such that *QaQ* ^−1^ = *a*′, *QbQ* ^−1^ = *b*′, *Q*(*ac*)*Q* ^−1^ = (*ac*)′, and *Q*(*cb*)*Q* ^−1^ = (*cb*)′ have all nonzero entries.

Notice that {*ac*, *a*} are linearly *Z*(*R*)-dependent if and only if {(*ac*)′, *a*′} are linearly *Z*(*R*)-dependent; analogously {*cb*, *b*} are linearly *Z*(*R*)-dependent if and only if {(*cb*)′, *b*′} are linearly *Z*(*R*)-dependent. Moreover *ac* = *cb* = 0 if and only if (*ac*)′ = (*cb*)′ = 0. Therefore, in order to prove our result, we may replace *a*,  *b*,  *ac*,  *cb*, respectively, by *a*′,  *b*′,  (*ac*)′,  (*cb*)′, so that *a*,  *b*,  *ac*, and *cb* have all nonzero entries.

For *x* = *e*_*ij*_ ∈ *S* we have

(37) $$\begin{array}{c} X=ace_{ij}b-ae_{ij}cb=0; \end{array}$$

in particular the (*j*, *i*)-entry of *X* is *q*_*ji*_ *b*_*ji*_ − *a*_*ji*_ *p*_*ji*_ = 0. Denote 0 ≠ *η* = *q*_*ji*_/*a*_*ji*_, so that *p*_*ji*_ = *ηb*_*ji*_. Let *I* be the identity matrix in *R* and *u* = *c* − *ηI*. Since *u* and *c* induce the same inner derivation in *R*, then *a*[*u*, *x*]*b* = 0; that is, *a*(*c* − *ηI*)*xb* − *ax*(*c* − *ηI*)*b* = 0, for all *x* ∈ *S*. Moreover *a* and *b* have all nonzero entries, and the (*j*, *i*)-entry of (*c* − *ηI*)*b* is zero. Thus we may apply Lemmas 12 and 13 and obtain *ac* = *ηa* and *cb* = *ηb*, as required.

**Proof of Proposition 4.**

If one assumes that *C* is infinite, the conclusion follows from Lemma 14.

Now let *K* be an infinite field which is an extension of the field *C* and let $\bar{R}=M_{t}(K)≅R{⊗}_{C}K$. Consider the generalized polynomial

(38) $$\begin{array}{c} P\left(x_{1},x_{2}\right)=a\left(c\left[x_{1},x_{2}\right]-\left[x_{1},x_{2}\right]c\right)b \end{array}$$

which is a generalized polynomial identity for *R*. Since *P*(*x*_1_, *x*_2_) is a multilinear generalized polynomial in the indeterminates *x*_1_,  *x*_2_, then it is a generalized polynomial identity for $\bar{R}$ and the conclusion follows again from Lemma 14.

## 3. The Inner-Case in Prime Rings

In this section we consider *f*(*R*), the set of all evaluations of the noncentral multilinear polynomial *f*(*x*_1_,…, *x*_*n*_) over *C*, and assume that *F* is an inner generalized derivation, so that there exist *c*, *q* ∈ *U* such that *F*(*x*) = *cx* + *xq*, for all *x* ∈ *R*, and *f*(*R*) satisfies

(39) $$\begin{array}{c} a\left[cx+xq,x\right]b, \end{array}$$

where *a*,  *b* are nonzero elements of *R*.

In order to prove the first result we premit the following.

**Fact 1 .**

Let *R* = *M*_*t*_(*C*) be the algebra of *t* × *t* matrices over *C* of characteristic different from 2. Notice that the set *f*(*R*) = {*f*(*r*_1_,…, *r*_*n*_) : *r*_1_,…, *r*_*n*_ ∈ *R*} is invariant under the action of all inner automorphisms of *R*. Hence if denoted by *r* = (*r*_1_,…, *r*_*n*_) ∈ *R* × *R* × *R* × ⋯×*R* = *R* ^*n*^, then for any inner automorphism *φ* of *M*_*t*_(*C*), we have that $\underline{r}=(\varphi (r_{1}),\ldots ,\varphi (r_{n}))\in R^{n}$ and $\varphi (f(r))=f(\underline{r})\in f(R)$.

Since *f*(*x*_1_,…, *x*_*n*_) is not central then, by [15] (see also [16]), there exist *u*_1_,…, *u*_*n*_ ∈ *M*_*t*_(*C*) and *α* ∈ *C* − {0}, such that *f*(*u*_1_,…, *u*_*n*_) = *αe*_*kl*_, with *k* ≠ *l*. Moreover, since the set {*f*(*v*_1_,…, *v*_*n*_) : *v*_1_,…, *v*_*n*_ ∈ *M*_*t*_(*C*)} is invariant under the action of all *C*-automorphisms of *M*_*t*_(*C*), then for any *i* ≠ *j* there exist *r*_1_,…, *r*_*n*_ ∈ *M*_*t*_(*C*) such that *f*(*r*_1_,…, *r*_*n*_) = *αe*_*ij*_.

Now we may start with the following.

**Proposition 15 .**

Let *C* be a field, *R* = *M*_*t*_(*C*) the algebra of *t* × *t* matrices over *C*, and *f*(*x*_1_,…, *x*_*n*_) a noncentral multilinear polynomial over *C*. Let 0 ≠ *a*,  0 ≠ *b*,  *c*, *q* ∈ *R* and denote

(40) $$\begin{array}{c} a=\underset{kl}{\sum }a_{kl}e_{kl},\;\;b=\underset{kl}{\sum }b_{kl}e_{kl},\\ c=\underset{kl}{\sum }c_{kl}e_{kl},\;\;q=\underset{kl}{\sum }q_{kl}e_{kl} \end{array}$$

for suitable *a*_*kl*_, *b*_*kl*_, *c*_*kl*_, and *q*_*kl*_ elements of *C*. Suppose that

(41) $$\begin{array}{c} a\left[cf\left(r_{1},\ldots ,r_{n}\right)+f\left(r_{1},\ldots ,r_{n}\right)q,f\left(r_{1},\ldots ,r_{n}\right)\right]b=0 \end{array}$$

for all *r*_1_,…, *r*_*n*_ ∈ *R*. Then one of the following holds:

(1) *c*, *q* ∈ *Z*(*R*);
(2) there exists *λ* ∈ *Z*(*R*) such that *c* − *q* = *λ*, and *f*(*x*_1_,…, *x*_*n*_)^2^ is central valued on *R*;
(3) there exist *λ*, *μ* ∈ *Z*(*R*) such that *c* − *q* = *λ*, *ac* = *μa* and *cb* = *μb*.

**Proof**

By our assumption, *R* satisfies the following generalized polynomial identity:

(42) $$\begin{array}{c} a\left[cf\left(x_{1},\ldots ,x_{n}\right)+f\left(x_{1},\ldots ,x_{n}\right)q,f\left(x_{1},\ldots ,x_{n}\right)\right]b. \end{array}$$

As in the previous section *e*_*ij*_ denotes the matrix unit with 1 in (*i*, *j*)-entry and zero elsewhere.

Firstly we assume *C* is an infinite field.

Since *f*(*x*_1_,…, *x*_*n*_) is not central then, by Fact 1, for any *i* ≠ *j*, there exist *r*_1_,…, *r*_*n*_ ∈ *M*_*t*_(*C*) such that *f*(*r*_1_,…, *r*_*n*_) = *e*_*ij*_.

Then we obtain

(43) $$\begin{array}{c} 0=a\left[ce_{ji}+e_{ji}q,e_{ji}\right]b=ae_{ji}qe_{ji}b-ae_{ji}ce_{ji}b. \end{array}$$

In particular,

(44) $$\begin{array}{c} a_{ij}\left(q_{ij}-c_{ij}\right)b_{ij}=0. \end{array}$$

In light of Remark 2, we assume that *a* and *b* are not central matrices. Denote *w* = *q* − *c* and suppose that *w* is not scalar. By Lemma 5 there exists an *C*-automorphism *φ* of *M*_*t*_(*C*) such that *w*′ = *φ*(*w*), *a*′ = *φ*(*a*), and *b*′ = *φ*(*b*) have all nonzero entries. Clearly *w*′, *a*′, and *b*′ must satisfy the condition (44) and this is a contradiction.

This means that *q* − *c* = *γI*, for some *γ* ∈ *C*, and the main condition is now

(45) $$\begin{array}{c} a\left[cf\left(r_{1},\ldots ,r_{n}\right)+f\left(r_{1},\ldots ,r_{n}\right)c,f\left(r_{1},\ldots ,r_{n}\right)\right]b=0, \end{array}$$

for all *r*_1_,…, *r*_*n*_ ∈ *R*; that is, *a*[*c*, *f*(*r*_1_,…, *r*_*n*_)^2^]*b* = 0, for all *r*_1_,…, *r*_*n*_ ∈ *R*.

Consider the additive subgroup of *R*, generated by the set *S* = {*x* ^2^ : *x* ∈ *f*(*R*)}. By [17], either *S*⊆*Z*(*R*) or the noncentral Lie ideal [*R*, *R*] of *R* is contained in *S*. In the first case we conclude that *f*(*x*_1_,…, *x*_*n*_)^2^ is central valued in *R* and we are done. In either case we have *a*[*c*, [*r*_1_, *r*_2_]]*b* = 0, for all *r*_1_, *r*_2_ ∈ *R*, and by Proposition 4 we get the required conclusions.

Now let *K* be an infinite field which is an extension of the field *C* and let $\bar{R}=M_{m}(K)≅R{⊗}_{C}K$. Notice that the multilinear polynomial *f*(*x*_1_,…, *x*_*n*_) is central-valued on *R* if and only if it is central-valued on $\bar{R}$. Consider the generalized polynomial

(46) Px1,…,xn=acfx1,…,xn2b−fx1,…,xn2qb ikx1,…,xn2+af(x1,…,xn)(q−c)f(x1,…,xn)b

which is a generalized polynomial identity for *R*. Moreover it is multihomogeneous of multidegree (2,…, 2) in the indeterminates *x*_1_,…, *x*_*n*_.

Hence the complete linearization of *P*(*x*_1_,…, *x*_*n*_) is a multilinear generalized polynomial Θ(*x*_1_,…, *x*_*n*_, *y*_1_,…, *y*_*n*_) in 2*n* indeterminates; moreover

(47) $$\begin{array}{c} \Theta \left(x_{1},\ldots ,x_{n},x_{1},\ldots ,x_{n}\right)=2^{n}P\left(x_{1},\ldots ,x_{n}\right). \end{array}$$

Clearly the multilinear polynomial Θ(*x*_1_,…, *x*_*n*_, *y*_1_,…, *y*_*n*_) is a generalized polynomial identity for *R* and $\bar{R}$ too. Since char(*C*) ≠ 2 we obtain *P*(*r*_1_,…, *r*_*n*_) = 0, for all $r_{1},\ldots ,r_{n}\in \bar{R}$, and the conclusion follows from the argument contained in the first part of this proposition.

**Lemma 16 .**

If there exist 0 ≠ *a* ∈ *R*, 0 ≠ *b* ∈ *R*, *c*, *q* ∈ *U* such that *a*[*cx* + *xq*, *x*]*b* = 0, for all *x* ∈ *f*(*R*), then *R* satisfies a nontrivial generalized polynomial identity, unless when one of the following holds:

(1) *c*, *q* ∈ *C*;
(2) *c* − *q* ∈ *C* and there exists *λ* ∈ *C* such that *ac* = *λa*,  *qc* = *λb*.

**Proof**

Assume that *R* does not satisfy any nontrivial generalized polynomial identity with coefficients in *U*. Therefore,

(48) Φx1,…,xn =acfx1,…,xn+fx1,…,xnq,fx1,…,xnb

is a trivial generalized polynomial identity for *R*. By calculations

(49) Φx1,…,xn=acfx1,…,xn2+fx1,…,xn ×q−cfx1,…,xn −fx1,…,xn2qb=0

for all *x*_1_,…, *x*_*n*_ ∈ *R*. If *c* ∈ *C* and *q* ∈ *C*, the proof is completed; hence we suppose that *c* and *q* are not simultaneously central. By Remark 3 and by (49), if {*b*, *qb*} are linearly *C*-independent then *R* satisfies the trivial generalized polynomial identity *af*(*x*_1_,…, *x*_*n*_)^2^ *qb* = 0. It means, since *a* ≠ 0, *qb* = 0, a contradiction. Analogously, if we suppose {*a*, *ac*} linearly *C*-independent, we get *ac* = 0, a contradiction.

Therefore there exist *α*, *β* ∈ *C* such that *qb* = *βb* and *ac* = *αa*; now (49) becomes

(50) af(x1,…,xn)(q−c)f(x1,…,xn)x1,…,xn2 +(α−β)fx1,…,xn2b=0

for all *x*_1_,…, *x*_*n*_ ∈ *R*. Since it is a trivial generalized polynomial identity, then *c* − *q* = *α* − *β*. Moreover, *βb* = *qb* = *cb* + *βb* − *αb*; that is, *cb* = *αb*.

**Proposition 17 .**

Let 0 ≠ *a*, 0 ≠ *b*, *c*, *q* ∈ *R* such that

(51) $$\begin{array}{c} a\left[cf\left(r_{1},\ldots ,r_{n}\right)+f\left(r_{1},\ldots ,r_{n}\right)q,f\left(r_{1},\ldots ,r_{n}\right)\right]b=0 \end{array}$$

for all *r*_1_,…, *r*_*n*_ ∈ *R*. Then one of the following holds:

(1) *c*, *q* ∈ *Z*(*R*);
(2) there exists *λ* ∈ *C* such that *c* − *q* = *λ*, and *f*(*x*_1_,…, *x*_*n*_)^2^ is central valued on *R*;
(3) there exist *λ*, *μ* ∈ *C* such that *c* − *q* = *λ*, *ac* = *μa*, and *cb* = *μb*.

**Proof**

By Remark 2 we assume that *R* is not a domain.

Moreover, by Lemma 16, *R* satisfies the nontrivial generalized polynomial identity:

(52) Px1,…,xn=acfx1,…,xn+fx1,…,xnq,kkkkfx1,…,xnb.

By a theorem due to Beidar (Theorem  2 in [18]) this generalized polynomial identity is also satisfied by *U*. In case *C* is infinite, we have *P*(*r*_1_,…, *r*_*n*_) = 0 for all $r_{1},\ldots ,r_{n}\in U{⊗}_{C}\bar{C}$, where $\bar{C}$ is the algebraic closure of *C*. Since both *U* and $U{⊗}_{C}\bar{C}$ are centrally closed [19, Theorems 2.5 and 3.5], we may replace *R* by *U* or $U{⊗}_{C}\bar{C}$ according to *C* being finite or infinite. Thus we may assume that *R* is centrally closed over *C* which is either finite or algebraically closed. By Martindale's theorem [14], *R* is a primitive ring having a nonzero socle *H* with *C* as the associated division ring, and *eHe* is a simple central algebra finite dimensional over *C*, for any minimal idempotent element *e* ∈ *RC*.

In light of Jacobson's theorem [20, page 75] *R* is isomorphic to a dense ring of linear transformations on some vector space *V* over *C*.

Assume first that *V* is finite-dimensional over *C*. Then the density of *R* on *V* implies that *R*≅*M*_*k*_(*C*), the ring of all *k* × *k* matrices over *C*. Since *R* is not commutative we assume *k* ≥ 2. In this case the conclusion follows by Proposition 15.

Assume next that *V* is infinite-dimensional over *C*. As in Lemma  2 in [21], the set *f*(*R*) is dense on *R* and so from *P*(*r*_1_,…, *r*_*n*_) = 0, for all *r*_1_,…, *r*_*n*_ ∈ *R*, we have that *R* satisfies the generalized identity *P*(*x*) = *a*[*cx* + *xq*, *x*]*b*. We remark that *H* satisfies *P*(*x*) = *a*(*cx* ^2^ − *x* ^2^ *q* + *x*(*q* − *c*)*x*)*b* = 0 (see, e.g., [5, proof of Theorem 1]); that is, for all *r* ∈ *H*,

(53) $$\begin{array}{c} a\left(cr^{2}-r^{2}q+r\left(q-c\right)r\right)b=0. \end{array}$$

In this equality we substitute *r* with *ex*(1 − *e*), for any nontrivial idempotent element *e* = *e* ^2^ ∈ *H*, and obtain

(54) $$\begin{array}{c} aex\left(1-e\right)\left(q-c\right)ex\left(1-e\right)b=0. \end{array}$$

By the primeness of *R*, it follows that either *ae* = 0 or (1 − *e*)*b* = 0 or (1 − *e*)(*q* − *c*)*e* = 0. Here our aim is to prove that in any case (1 − *e*)(*q* − *c*)*e* = 0. To do this, we firstly assume that *ae* = 0. In (53) replace *r* by *ex*, so that *ac*(*ex*)^2^ *b* = 0, which implies *ace* = 0.

Moreover we substitute in (53)*r* with *ex* + *y*(1 − *e*) and by easy computation it follows *ay*(1 − *e*)(*q* − *c*)*exb* = 0; that is, (1 − *e*)(*q* − *c*)*e* = 0.

On the other hand, if one supposes (1 − *e*)*b* = 0 and replacing in (53)*r* by *x*(1 − *e*), one has *a*(*x*(1 − *e*))^2^ *qb* = 0, which implies (1 − *e*)*qb* = 0. Finally, if substituted in (53)*r* with *x*(1 − *e*) + *ey*, as above we have *ax*(1 − *e*)(*q* − *c*)*eyb* = 0. Thus in any case it follows (1 − *e*)(*q* − *c*)*e* = 0.

Similarly one can prove also that *e*(*q* − *c*)(1 − *e*) = 0.

Hence [*q* − *c*, *e*] = 0, for any idempotent element *e* ∈ *H*. Since *H* is not a domain, then *H* is generated by its minimal idempotent elements; therefore [*q* − *c*, *H*] = (0); that is, *q* − *c* ∈ *C*. Let *λ* ∈ *C* such that *q* = *c* + *λ*. By our assumption it follows that *H* satisfies *a*[*cx* + *xc*, *x*]*b* that is *H* satisfies *a*[*c*, *x* ^2^]*b*. In this last replace *x* by *x* + 1 and obtain that *H* satisfies *a*[*c*, 2*x*]*b*. Since char(*H*) ≠ 2, then *acrb* − *arcb* = 0, for all *r* ∈ *H*. By [14, Lemma 1] it follows that there exists *μ* ∈ *C* such that *ac* = *μa* and *cb* = *μb*, unless *ac* = *cb* = 0.

**Corollary 18 .**

Let *a*, *b*, *c* ∈ *R* such that *c* ∉ *C* and *f*(*x*_1_,…, *x*_*n*_) be a noncentral multilinear polynomial over *C*. If *a*[*c*, *f*(*r*_1_,…, *r*_*n*_)]_2_ *b* = 0, for all *r*_1_,…, *r*_*n*_ ∈ *R*, then either *a* = 0 or *b* = 0.

## 4. The Main Result

In [11] Lee proved that every generalized derivation can be uniquely extended to a generalized derivation of *U* and thus all generalized derivations of *R* will be implicitly assumed to be defined on the whole *U* and obtained the following result.

**Theorem 19 (Theorem 3 in [11]).**

Every generalized derivation *F* on a dense right ideal of *R* can be uniquely extended to *U* and assumes the form *F*(*x*) = *cx* + *d*(*x*), for some *c* ∈ *U* and a derivation *d* on *U*.

In this section we denote by *f* ^*d*^(*x*_1_,…, *x*_*n*_) the polynomial obtained from *f*(*x*_1_,…, *x*_*n*_) by replacing each coefficient *α*_*σ*_ with *d*(*α*_*σ*_). Thus we write *d*(*f*(*r*_1_,…, *r*_*n*_)) = *f* ^*d*^(*r*_1_,…, *r*_*n*_) + ∑_*i*_ *f*(*r*_1_,…, *d*(*r*_*i*_),…, *r*_*n*_), for all *r*_1_,…, *r*_*n*_ in *R*.

In light of this, we finally prove our main result.

**Proof of Theorem 1.**

Suppose both *a* ≠ 0 and *b* ≠ 0. Since *R* satisfies the generalized differential identity

(55) $$\begin{array}{c} a\left[F\left(f\left(x_{1},\ldots ,x_{n}\right)\right),f\left(x_{1},\ldots ,x_{n}\right)\right]b, \end{array}$$

the above cited Lee's result says that *R* satisfies

(56) $$\begin{array}{c} a\left[cf\left(x_{1},\ldots ,x_{n}\right)+d\left(f\left(x_{1},\ldots ,x_{n}\right)\right),f\left(x_{1},\ldots ,x_{n}\right)\right]b. \end{array}$$

If *d* is an inner derivation induced by an element *q* ∈ *U*, then *R* satisfies the generalized polynomial identity:

(57) acfx1,…,xn+qfx1,…,xn−fx1,…,xnq,kkkfx1,…,xnb

which is

(58) $$\begin{array}{c} a\left[\left(c+q\right)f\left(x_{1},\ldots ,x_{n}\right)-f\left(x_{1},\ldots ,x_{n}\right)q,f\left(x_{1},\ldots ,x_{n}\right)\right]b. \end{array}$$

In this case we are done by Proposition 17.

Hence let *d* be an outer derivation of *R*. In this case *R* satisfies the differential identity:

(59) acf(x1,…,xn)+fd(x1,…,xn)∑ikk+∑if(x1,…,d(xi),…,xn),kkk∑if(x1,….,xn)b.

By Kharchenko's theorem (see [16, 22]), *R* satisfies the generalized polynomial identity:

(60) acfx1,…,xn+fdx1,…,xn∑ikkk+∑ifx1,…,yi,…,xn,kkkk∑ifx1,…,xnb,

and in particular, for all *i* = 1,…, *n*, *R* satisfies the blended component

(61) $$\begin{array}{c} a\left[f\left(x_{1},\ldots ,y_{i},\ldots ,x_{n}\right),f\left(x_{1},\ldots ,x_{n}\right)\right]b. \end{array}$$

Let *q* ∈ *R* − *Z*(*R*) and replace any *y*_*i*_ by [*q*, *x*_*i*_]. Thus *R* satisfies

(62) $$\begin{array}{c} a\left[\underset{i}{\sum }f\left(x_{1},\ldots ,\left[q,x_{i}\right],\ldots ,x_{n}\right),f\left(x_{1},\ldots ,x_{n}\right)\right]b. \end{array}$$

that is,

(63) $$\begin{array}{c} a{\left[q,f\left(x_{1},\ldots ,x_{n}\right)\right]}_{2}b. \end{array}$$

By Corollary 18, we get the contradiction *q* ∈ *Z*(*R*).
